# Unique Clinical Features of Imaging-Stage I Peripheral Lung Squamous Cell Carcinoma: A Retrospective Study

**DOI:** 10.3390/curroncol33010047

**Published:** 2026-01-15

**Authors:** Chengzhang Xiong, Wenjing Zhang, Qing Wang, Hao Yin, Jibin Chen, Wenjun Jiang, Xu Han

**Affiliations:** Department of Thoracic Surgery, The Fourth Affiliated Hospital of China Medical University, Shenyang 110032, China

**Keywords:** peripheral lung squamous cell carcinoma, prognostic factor, computed tomography, volume doubling time

## Abstract

The incidence of peripheral lung squamous cell carcinoma (p-LUSC) has increased in recent years, but the clinical features of early-stage p-LUSC remain unclear. In the present study, we aim to elucidate the general clinical features of p-LUSC by comparing it with peripheral lung adenocarcinoma (p-LUAD). Patients with p-LUSC or p-LUAD who were at an early imaging stage and underwent complete lobectomy with systematic lymph node dissection were included. The clinical characteristics of p-LUSC were elucidated through comparative analysis with p-LUAD, and independent prognostic factors for recurrence-free survival were identified. A total of 103 patients with p-LUSC and 600 patients with p-LUAD were included. Compared with p-LUAD, all p-LUSC cases appeared as solid nodules (SDNs) on imaging, and p-LUSC was associated with the male sex, older age, smoking history, lobulation sign, interstitial pneumonia, and a shorter volume doubling time. In terms of malignant aggressiveness, p-LUSC demonstrated a significantly lower lymph node metastasis rate than SDNs of p-LUAD in the >2.0 to ≤3.0 cm group, while no statistically significant difference was observed between the two groups in the 0–2.0 cm group. As for prognosis, tumor size and lymph node metastasis were found as independent risk factors for tumor recurrence.

## Simple Summary

Although the incidence of peripheral lung squamous cell carcinoma (p-LUSC) has increased in recent years, the clinical features of early-stage p-LUSC remain unclear. We conducted a retrospective analysis of 103 cases of p-LUSC and 600 cases of peripheral lung adenocarcinoma, revealing that p-LUSC exhibited distinct characteristics in terms of patient demographics, imaging presentation, clinicopathological features, and tumor volume doubling time. A systematic summary of these unique clinical features in early-stage p-LUSC fosters heightened clinician vigilance before pathological confirmation. Additionally, the relatively low malignant invasiveness observed in >2.0 to ≤3.0 cm p-LUSC tumors offered promising directions for future research on personalized treatment strategies.

## 1. Introduction

Lung cancer is the most prevalent malignant tumor and the leading cause of tumor mortality [1]. Although the incidence is lower than that of lung adenocarcinoma (LUAD), lung squamous cell carcinoma (LUSC) remains one of the most common types of lung cancer [2]. LUSC is classified into central LUSC (c-LUSC) and peripheral LUSC (p-LUSC) according to the primary tumor site, with the boundary defined by the segmental bronchus [3]. It was a commonly held belief that the majority of LUSC cases were c-LUSC; however, recent reports have identified a higher incidence of p-LUSC [4,5]. Despite this, further clinical studies remain scarce, particularly those focusing on early-stage p-LUSC.

The prevailing understanding of the clinical features of early-stage peripheral non-small cell lung cancer (NSCLC) has predominantly concerned p-LUAD due to its high prevalence. A substantial proportion of early p-LUAD appears as ground-glass nodules (GGNs) on chest CT scans, and the consolidation/tumor ratio (CTR) and tumor size have been identified as the two most crucial indicators for predicting the malignancy [6,7]. However, research on the imaging features of early-stage p-LUSC is limited, and even less is known regarding the clinical implications of these findings. Moreover, while a number of studies have indicated a correlation between malignant aggressiveness and tumor size in early p-LUAD [8,9], this relationship in p-LUSC remains indeterminate. Research on LUSC suggests that early-stage p-LUSC may exhibit distinct characteristics compared to p-LUAD [10]. Thus, we focused on imaging early-stage p-LUSC and emphasized its clinical features by comparing them with those of p-LUAD.

## 2. Materials and Methods

### 2.1. Patient Cohorts

Cases of p-LUSC and p-LUAD that were surgically resected and pathologically confirmed from January 2016 to January 2022 at the Department of Thoracic Surgery of the Fourth Affiliated Hospital of China Medical University were collected. Peripheral pulmonary tumors (p-LUSC and p-LUAD) were defined as lesions originating from subsegmental or more distal bronchi and bronchioles, as identified on chest CT imaging [3]. The inclusion criteria were as follows: (1) preoperative imaging requirements: head CT, chest high-resolution CT (HRCT) or contrast-enhanced CT, and abdominal–pelvic CT scans performed within 1 week prior to surgery; (2) a single lesion without evidence of distant metastasis; (3) a maximum nodule diameter less than 3 cm and short-axis diameters of hilar and mediastinal lymph nodes less than 1 cm on imaging; (4) surgical resection (lobectomy or segmentectomy) with systematic lymph node dissection; and (5) postoperative histopathological confirmation of primary lung cancer.

### 2.2. General Clinical Data

We reviewed the hospital’s electronic medical record system to collect demographic and clinical data, including age, gender, smoking history, medical history, and postoperative complications. Tumor marker levels were also extracted. Preoperatively identified high-risk patients included those with any of the following (as reported in ref. [11]): pulmonary dysfunction, a history of myocardial infarction or angina pectoris, a history of cerebral infarction, or insulin-dependent diabetes mellitus. Tumor markers included ferritin, carcinoembryonic antigen (CEA), carbohydrate antigen 19-9 (CA19-9), and cytokeratin 19 fragment (CYFRA21-1). The institutional reference ranges for these tumor markers were as follows: 22–322 ng/mL, 0–5 ng/mL, 0–37 U/mL, and 0.1–3.3 ng/mL, respectively.

### 2.3. Imaging Data

**Figure 1:**
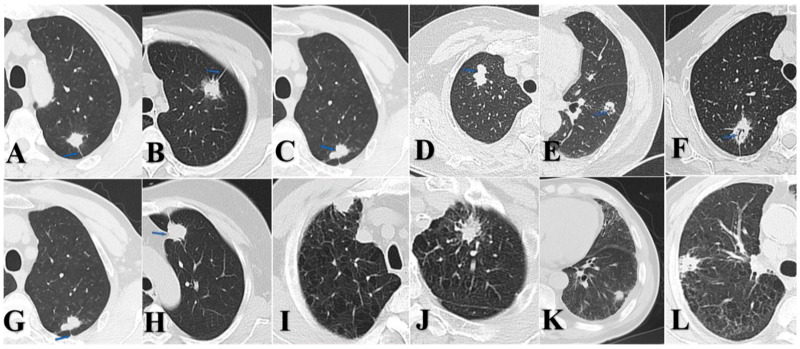
(**A**) p-LUSC, male, spicular sign. (**B**) p-LUAD, female, spicular sign. (**C**) p-LUSC, male, lobulation sign. (**D**) p-LUAD, female, lobulation sign. (**E**) p-LUSC, male, air bronchogram. (**F**) p-LUAD, female, air bronchogram. (**G**) p-LUSC, male, pleural indentation. (**H**) p-LUAD, female, pleural indentation. (**I**) p-LUSC, male, emphysema. (**J**) p-LUAD, male, emphysema. (**K**) p-LUSC, male, interstitial pneumonia. (**L**) p-LUAD, male, interstitial pneumonia.

Two board-certified radiologists blinded to pathological results independently assessed the consolidation tumor ratio (CTR) for each case. Based on CTR values, all lesions were categorized as follows: solid nodules (SDNs, CTR = 100%), mixed ground-glass nodules (mGGNs, CTR ≥ 50%), and mGGNs CTR < 50%. Both radiologists independently extracted the following imaging features (Figure 1): spicular sign, lobulation sign, air bronchogram, pleural indentation, pulmonary emphysema, and interstitial pneumonia. Final determinations were reached through consensus in cases of disagreement.

Tumor volume reconstruction was conducted by a radiologist using dedicated software for patients with either p-LUSC or p-LUAD who had documented follow-up data with intervals exceeding 3 months before surgery. Volume doubling time (VDT) was calculated using the formula from ref. [12]: VDT = (t∗ln2)/[ln(V1/V0)], where “t” represents the time interval between two follow-up assessments, “V1” denotes the tumor volume at the second follow-up, and “V0” represents the tumor volume at the first follow-up.

### 2.4. Follow-Up and Endpoints

Cases with systematic postoperative follow-up were included. Postoperative surveillance required outpatient thoracic surgery consultations and chest CT scans at least every 6 months during the first 2 postoperative years. Beyond 2 years, follow-up intervals were adjusted to annual outpatient thoracic surgery consultations and chest CT scans. Relapse-Free Survival (RFS) represented the duration from pathological diagnosis of lung cancer to locoregional recurrence, distant metastasis, or last follow-up. For patients with suspected recurrence on imaging studies, clinical recurrence was diagnosed through multidisciplinary discussion involving at least one radiologist and one thoracic surgeon, or confirmed pathologically via biopsy.

### 2.5. Statistical Analysis

Categorical clinical variables were analyzed using the chi-squared test or Fisher’s exact test as appropriate. Continuous variables were analyzed using *t*-tests if normally distributed (assessed by Shapiro–Wilk normality tests); otherwise, they were analyzed using the Mann–Whitney U test. Variables with a *p*-value less than 0.1 were further included in logistic regression analysis to identify independent factors capable of distinguishing between p-LUSC and p-LUAD. The discrimination was evaluated using the integrated area under the receiver operating characteristic curves (iAUCs) for independent factors.

The probability of the RFS rate was estimated via the Kaplan–Meier method, while differences in survival rates between groups in the univariate comparison were analyzed using the log-rank test. Variables with a *p*-value less than 0.1 were further incorporated into the Cox proportional hazards model to identify independent prognostic predictors. Statistical analyses were performed using SPSS version 27.0 (SPSS Inc., Chicago, IL, USA) with statistical significance defined as two-sided *p* ≤ 0.05.

## 3. Results

### 3.1. Patient Baseline Characteristics

A total of 103 p-LUSC and 600 p-LUAD patients were included. The baseline characteristics are presented in Table 1. The median age was 68 years for p-LUSC and 62 years for p-LUAD. Lymph node metastasis was observed in 59 cases, accounting for 8.4% of the total cohort.

Statistical differences were observed between the p-LUSC and p-LUAD in terms of gender ratio (86.4% vs. 40%, *p* < 0.001), median age (68 vs. 62, *p* < 0.001), tumor size (51.5% vs. 66.2%, *p* = 0.0041), smoking history (55.3% vs. 17.5%, *p* < 0.001), high surgical risk (36.9% vs. 18.8%, *p* < 0.001), and imaging CTR (100% vs. 36%, *p* < 0.001). However, no statistically significant difference was observed for lymph node metastasis rates. Notably, all p-LUSC cases were SDNs on CT scan, while only 36% of p-LUAD were SDNs.

### 3.2. General Characteristics of p-LUSC and SDNs of p-LUAD Patients

Given the fundamental difference found on CT scans, p-LUAD cases presenting GGNs (CTR < 50% and CTR ≥ 50%) were excluded from further study, and a total of 103 p-LUSC cases and 216 p-LUAD cases presenting as SDNs were incorporated instead (Table 2). Compared with SDNs of p-LUAD, p-LUSC was found to be associated with the male sex (86.4% vs. 54.2%, *p* < 0.001), older age (68 vs. 63, *p* < 0.001), smoking history (55.3% vs. 27.3%, *p* = 0.003), high surgical risk (36.9% vs. 22.2%, *p* = 0.0058), and higher frequencies of lobulation (73.8% vs. 31.0%, *p* < 0.001), emphysema (14.6% vs. 3.7%, *p* < 0.001), and interstitial pneumonia (24.3% vs. 8.8%, *p* < 0.001). In terms of tumor markers, p-LUSC was found to be higher in CYFRA21-1 (2.0 vs. 1.8, *p* = 0.024).

**Figure 2:**
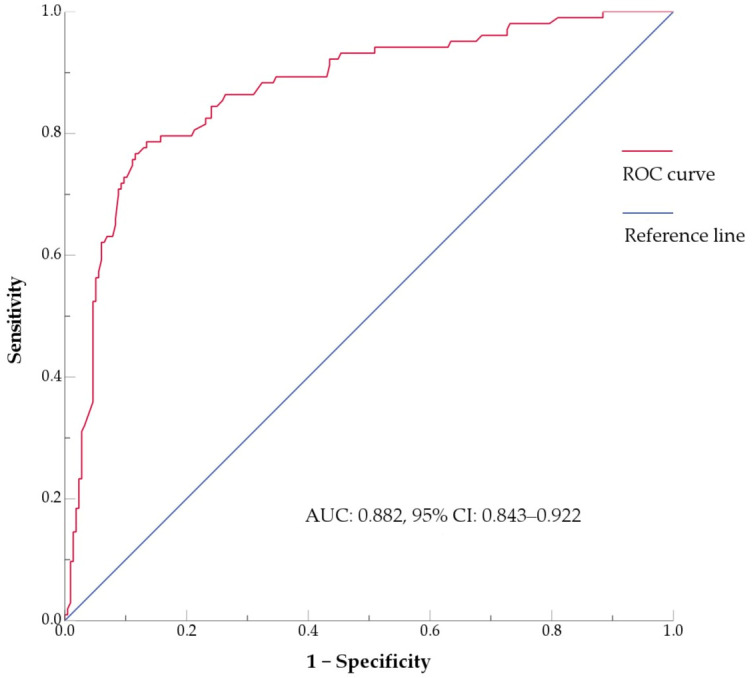
ROC curve of the multivariate model for p-LUSC incorporating gender, age, smoking history, lobulation sign, and interstitial pneumonia. AUC, area under the curve; CI, confidence interval; ROC, receiver operating characteristic.

Further logistic regression analysis showed that gender ratio (*p* < 0.001), age (*p* < 0.001), smoking history (*p* = 0.004), lobulation sign (*p* < 0.001), and interstitial pneumonia (*p* = 0.019) were independent factors. The area under the receiver operating characteristic curve (AUC) values for the evaluated factors were as follows: gender ratio (0.661, 95% CI: 0.600–0.722), age (0.725, 95% CI: 0.665–0.784), smoking history (0.640, 95% CI: 0.574–0.706), lobulation sign (0.714, 95% CI: 0.653–0.775), and interstitial pneumonia (0.577, 95% CI: 0.508–0.647). The combined predictive model integrating these factors (Figure 2) achieved an AUC of 0.882 (95% CI: 0.843–0.922).

Tumor doubling time was calculated for 62 cases of p-LUSC and 106 cases of p-LUAD presenting SDNs for whom preoperative follow-up records were available. We found that the VDT for p-LUSC ranged from 95.7 to 199.1 days, with a median of 134.3 days, whereas the VDT for SDNs of p-LUAD ranged from 231.4 to 646.8 days, with a median of 399.7 days (*p* = 0.002).

### 3.3. Clinicopathological Characteristics of p-LUSC and SDNs of p-LUAD Patients

Further grouping based on tumor size (Table 3 and Table 4) revealed that compared with >2.0 to ≤3.0 cm p-LUSC, p-LUAD presenting SDNs was more malignantly aggressive in lymph node metastasis (41.6% vs. 6%, *p* < 0.001) and pleural invasion (48.3% vs. 6%, *p* < 0.001). Compared with the group of 0–2.0 cm SDNs of p-LUAD, the >2.0 to ≤3.0 cm SDNs of p-LUAD group had a higher positivity rate of lymph node metastasis (41.6% vs. 7.1%, *p* < 0.001), pleural invasion (48.3% vs. 21.3%, *p* < 0.001), and lymphovascular invasion (34.8% vs. 11.0%, *p* < 0.001), while no statistical difference was found in lymph node metastasis, pleural invasion, or lymphovascular invasion between the 0–2.0 cm and >2.0 to ≤3.0 cm p-LUSC.

### 3.4. Identification of Independent Prognostic Factors for RFS

A total of 103 cases of p-LUSC and 216 cases of p-LUAD were included in the prognostic analysis. Among them, 24 patients had a follow-up duration of less than 36 months, resulting in a loss-to-follow-up rate of 7.5%. The median follow-up time was 39 months for p-LUSC and 40 months for p-LUAD. The overall 3-year RFS rate was 88.1%. The tumor size (HR: 3.507, 95%CI: (1.738–7.076), *p* < 0.001), lymph node metastasis (HR: 3.924, 95%CI: (2.046–7.524), *p* < 0.001), ferritin (HR: 1.003, 95%CI: (1.001–1.005), *p* = 0.003), and pleural invasion (HR: 2.262, 95%CI: (1.180–4.338), *p* = 0.014) were all found to be associated with prognosis, as shown in Table 5.

Variables with a *p*-value less than 0.1 in the univariate analysis were included in the multivariate Cox regression analysis. Tumor size (HR: 2.430, 95%CI: (1.135–5.204), *p* = 0.022) and lymph node metastasis (HR: 2.305, 95%CI: (1.083–4.903), *p* < 0.001) were identified as independent prognostic factors.

## 4. Discussion

While the central type has traditionally been regarded as the primary form of LUSC, recent studies have reported an increasing incidence of the peripheral type, with some research even indicating that peripheral squamous cell carcinoma has become more common. A study from Taiwan analyzing data from 1990 to 2013 found that p-LUSC comprised 44.7% of all LUSC cases [13]. Similarly, a Japanese study reviewing patients who underwent lung cancer surgery between 2003 and 2019 reported that p-LUSC accounted for 64% of clinical-stage I LUSC cases [5]. Supporting this trend, a study conducted in Maryland, USA, which reviewed newly diagnosed LUSC cases from 2012 to 2013 at a central hospital in Baltimore, revealed that p-LUSC constituted 55% of new LUSC cases. Furthermore, when patients with a prior history of malignancy were excluded, the proportion of p-LUSC among new cases rose to 62.1% [4]. These findings collectively suggest a notably high proportion of p-LUSC in the current landscape of LUSC incidence. Our study did not consider c-LUSC but focused on radiologically early p-LUSC and p-LUAD. We found that the ratio of new p-LUSC to p-LUAD cases was approximately 1:6, which aligns reasonably well with previously published data. Unfortunately, these findings do not allow for the determination of the exact incidence rate of p-LUSC. Moreover, large-scale, high-quality epidemiological studies on lung cancer generally do not report the incidence of p-LUSC separately.

In this study, we found that patients with p-LUSC were significantly older at diagnosis compared to those with p-LUAD. Additionally, p-LUSC was associated with a higher prevalence of male sex and a greater proportion of current or former smokers. This aligns with the findings of Yang et al., who reported that LUSC patients exhibited a higher proportion of older individuals, male sex, and current or former smokers compared with those with LUAD [14]. Our results also suggest that the previously identified demographic characteristics remain relevant in early-stage p-LUSC. Sakurai et al. reported that 56% of patients with p-LUSC ≤ 3 cm were classified as high surgical risk due to advanced age and comorbidities [11]. In the present study, 36.9% of p-LUSC cases were defined as high surgical risk, which was significantly higher than that observed in p-LUAD (36.9% vs. 18.8%, *p* < 0.001). When further compared with SDNs of p-LUAD, the proportion of patients defined as high surgical risk in p-LUSC was also higher (36.9% vs. 22.2%, *p* = 0.0058).

Tumor markers play a crucial role in lung cancer screening and diagnosis. Commonly used markers include CEA, CYFRA 21-1, NSE, CA19-9, and ferritin [15,16]. In the present study, we found that these tumor markers exhibited low sensitivity for early-stage p-LUSC and p-LUAD. Compared with SDNs of p-LUAD, p-LUSC demonstrated elevated positivity for CYFRA 21-1, which was a soluble cytokeratin 19 fragment expressed in bronchial epithelium and related malignancies, released during cell degradation, and thus represents a useful serum tumor marker. Fu et al. demonstrated that CYFRA21-1 testing exhibited superior diagnostic accuracy for LUSC compared to adenocarcinoma [17]. Our study demonstrated significantly higher CYFRA21-1 levels in early-stage p-LUSC compared to p-LUAD. However, the diagnostic utility of CYFRA21-1 may require further validation due to the limited cohort size in this investigation.

The manifestations of malignant tumors on CT scans play a significant role in diagnosis [18]. In this study, all early-stage p-LUSC presented as SDNs on imaging, whereas 64% of p-LUAD manifested as GGNs. This observation contrasts with Akata’s findings, where one case of GGN was identified among 27 p-LUSC cases measuring < 2 cm. But pathological examination attributed the ground-glass opacity to inflammatory changes rather than tumor tissue [19]. A number of case reports have documented p-LUSC presenting as GGNs on chest CT scans [20,21]. Nevertheless, our study found that all 103 p-LUSC cases exhibited as SDNs, suggesting that GGN presentation in p-LUSC is relatively uncommon.

Akata et al. reported that the lobulation sign was present in 29.6% of p-LUSC lesions < 2 cm [19], whereas our study found a significantly higher prevalence of the lobulation sign in p-LUSC (73.8%) compared to SDNs of p-LUAD (31.0%). Furthermore, multivariate analysis confirmed the lobulation sign as an independent diagnostic factor for p-LUSC. Smith et al. found CT-detected emphysema in 70.4% of LUSC cases and established a significant association between emphysema and LUSC risk [22]. In our cohort, 14.6% of p-LUSC cases exhibited emphysema—higher than the 3.7% rate in p-LUAD. While Hubbard et al. reported increased lung cancer incidence in patients with interstitial fibrosis [23], we observed interstitial pneumonia in 24.3% of p-LUSC cases, which is significantly higher than the 8.8% in p-LUAD. Multivariable analysis identified interstitial pneumonia as an independent predictor, but not emphysema.

Previous studies have demonstrated shorter tumor doubling times in LUSC compared to LUAD [24,25]. Wilson DO et al. found that the median doubling time for LUSC was 160 days, which is faster than the 387 days for LUAD [24]. Mackintosh JA et al. reported that the median doubling time was 97 days for LUSC and 249 days for LUAD [25]. There has been little research on the tumor growth characteristics of early-stage p-LUSC and p-LUAD. Our study confirms for the first time that p-LUSC exhibited a significantly shorter doubling time (95.7–199.1 days, median = 134.3) compared to SDNs of p-LUAD (231.4–648.4 days, median = 399.7; *p* = 0.002). Our results are consistent with previous studies, suggesting that the characteristic of faster growth rate in LUSC may be present across different tumor sites or stages. This accelerated proliferation rate in p-LUSC has two important implications: it underscores the need for more aggressive follow-up protocols and treatment strategies, and the faster growth rate may serve as a valuable diagnostic criterion for differentiating SDNs of p-LUAD from p-LUSC. Notably, our study was limited by incomplete preoperative imaging follow-up data for some patients, which precluded multivariate analysis. Interestingly, we observed that p-LUSC doubling times were narrowly distributed, contrasting with the wider range seen in p-LUAD [26]. This concentration of doubling times suggests that the rapid growth of p-LUSC may be driven by specific oncogenic signaling pathways. This hypothesis is supported by Abraham et al., who identified inactivation of RHOA and TGFBR2 receptor, and ΔNp63α overexpression in >80% of LUSC cases [27], which was associated with proliferation and rarely observed in LUAD [28]. These findings point to potential molecular mechanisms underlying the observed differences in tumor growth rate.

In LUAD, a shorter VDT often indicates more aggressive histological subtypes with poorer prognosis, such as the solid or micropapillary patterns [29]. This implies that faster-growing malignant lung tumors should possess higher rates of lymph node metastasis, lymphovascular invasion, and pleural invasion. However, previous studies, along with our own findings, do not support this assumption. We found that p-LUSC with a faster proliferation rate demonstrated lower invasive potential. Similarly, a study by Mackintosh JA found three cases of slow-growing LUAD that nevertheless developed distant metastases [25]. In fact, this counterintuitive phenomenon cannot be readily explained by study bias alone. Compared with LUAD, the characteristics of LUSC—a shorter tumor doubling time yet lower invasiveness—have been observed in a number of previous studies [24,30,31]. This indicates that the tumor growth rate itself does not completely correlate with higher malignant aggressiveness. This intriguing manifestation is more likely an inherent characteristic of LUSC itself [32,33].

Our subgroup analysis—stratified by tumor size and CTR—revealed that >2.0 to ≤3.0 cm SDNs of p-LUAD demonstrated significantly greater malignant invasion than both 0–2.0 cm SDNs of p-LUAD and >2.0 to ≤3.0 cm p-LUSC. Notably, no statistically significant difference in lymph node metastasis was observed between >2.0 and ≤3.0 cm and 0–2.0 cm p-LUSC cases, which is consistent with the established literature [10,34]. Our study revealed that p-LUSC exhibits a distinct behavior: it rarely presents as GGNs, and the malignant aggressiveness does not increase with a tumor diameter up to 3 cm. Of note, in the 0–2.0 cm subgroup, p-LUSC and SDNs of p-LUAD showed no statistically significant differences in lymph node metastasis and lymphovascular invasion; however, p-LUAD demonstrated greater aggressiveness in terms of pleural invasion. In the >2.0 to ≤3.0 cm subgroup, SDNs of p-LUAD exhibited a more pronounced propensity for pleural invasion, whereas p-LUSC did not. This observation is consistent with previous reports; meanwhile, LUAD appears to have a propensity for pleural seeding after invading the pleura [35,36,37,38]. Even when pleural invasion occurs, LUSC tends to directly invade the chest wall rather than cause extensive pleural seeding metastasis or malignant pleural effusion [39,40]. Figure 1I demonstrates a preoperative CT scan of a patient with p-LUSC at our institution, revealing a mass located in the right upper lobe of the lung, abutting the pleura, with manifestations of pleural thickening, pleural involvement, without signs of pleural dissemination, malignant pleural effusion, or distant metastasis. Postoperative pathology confirmed visceral pleural invasion and desmoplastic reaction. This suggests that p-LUSC, when invading the pleura, is often accompanied by desmoplastic reaction, which leads to a tendency for local invasion rather than distant dissemination. These clinically relevant characteristics of early-stage p-LUSC have been underemphasized in the previous literature, despite its significant implications for clinical management.

Sublobar resection for early-stage p-NSCLC has garnered increasing attention and clinical application [41,42,43,44]. This study revealed that imaging early-stage p-LUSC and p-LUAD with a tumor diameter of 0–2.0 cm exhibited lower malignant invasiveness and superior prognoses compared to the >2.0 to ≤3.0 cm subgroup, which aligns with the aforementioned conclusions. Furthermore, Kamigaichi A et al. initiated a phase III trial comparing anatomical segmentectomy and lobectomy for clinical-stage IA3 pure-solid NSCLC [44]. We look forward to the final results of this study being reported. However, our findings demonstrated that the >2.0 to ≤3.0 cm subgroup exhibited worse prognosis. Notably, many p-LUAD patients with tumors measuring >2.0 to ≤3.0 cm showed no obvious lymph node metastasis on preoperative imaging, yet postoperative pathology confirmed the presence of lymph node metastasis. These findings may pose significant challenges to the successful progression of this study. Our study also suggests that, compared with p-LUAD, p-LUSC measuring >2.0 to ≤3.0 cm may be more suitable candidates for sublobar resection.

Multivariate Cox prognostic analysis for both p-LUSC and p-LUAD identified tumor size (0–2.0 cm vs. >2.0 to ≤3.0 cm) and lymph node metastasis as independent prognostic factors, which is consistent with the current TNM staging system for lung cancer. None of the other variables included in this study—such as pathological types [45,46], pleural invasion and lymphovascular invasion—were identified as independently associated with prognosis. However, it has to be acknowledged that this study was retrospective, with a follow-up period of only 3 years.

In the absence of a pathological biopsy, p-LUSC is difficult to identify with certainty from SDNs of p-LUAD. Even in some cases, in the absence of immunohistochemical staining, the intraoperative frozen section was difficult to identify as well, which hindered further personalized surgical option. In this study, logistic regression analysis revealed gender, age, smoking history, lobulation sign, and interstitial pneumonia as independent influencing factors, which could help in clinical differentiation. Since >2.0 to ≤3.0 cm SDNs of p-LUAD were significantly more invasive than p-LUSC, relying on preoperative data for differential diagnosis was meaningful for surgical planning.

This study revealed the unique clinical characteristics of p-LUSC. However, the following limitations should be considered: first, this was a single-center retrospective study with a limited sample size of p-LUSC cases; and second, some patients lacked preoperative follow-up records, resulting in missing VDT data.

## 5. Conclusions

The clinical features of p-LUSC are markedly different from p-LUAD, with early-stage p-LUSC almost always presenting as SDNs, and, for p-LUSC ≤3.0 cm, malignant aggressiveness does not increase with increasing tumor diameter. For 0–2.0 cm tumors, the clinicopathological features of p-LUSC are similar to p-LUAD, but for >2.0 to ≤3.0 cm, the features of p-LUSC are much less invasive. Compared to p-LUAD, the rate of proliferation of p-LUSC was much faster and more homogeneous. Although p-LUSC and p-LUAD exhibit similar imaging presentations, we identified certain clinical characteristics that can be utilized for differential diagnosis prior to pathological confirmation, including gender, age, smoking history, lobulation sign, interstitial pneumonia, and tumor doubling time. These insights are highly relevant for devising individualized clinical treatment regimens.

## Figures and Tables

**Table 1:** Patient characteristics (*n* = 703).

Factors	Total, *n* = 703	p-LUSC, *n* = 103	p-LUAD, *n* = 600	*p* Value
Gender				<0.001
Females	374 (53.2%)	14 (13.6%)	360 (60%)	
Males	329 (46.8%)	89 (86.4%)	240 (40%)	
Age, years				<0.001
Median (range), years	63 (21–84)	68 (47–84)	62 (21–83)	
Tumor size				0.0041
0–2.0 cm	450 (64.0%)	53 (51.5%)	397 (66.2%)	
>2.0 to ≤3.0 cm	253 (36.0%)	50 (48.5%)	203 (33.8%)	
Smoking history				<0.001
Current or former	162 (23.0%)	57 (55.3%)	105 (17.5%)	
Never	541 (77.0%)	46 (44.7%)	495 (82.5%)	
High surgical risk				<0.001
Yes	165 (23.5%)	38 (36.9%)	113 (18.8%)	
No	538 (76.5%)	65 (63.1%)	487 (81.2%)	
Lymph node metastasis				0.31
pN1 + pN2	59 (8.4%)	6 (5.8%)	53 (8.8%)	
pN0	644 (91.6%)	97 (94.2%)	547 (91.2%)	
Imaging CTR				<0.001
SDNs	319 (45.4%)	103 (100%)	216 (36%)	
mGGNs, CTR ≧ 50%	222 (31.6%)	0 (0%)	222 (37%)	
mGGNs, CTR < 50%	162 (23.0%)	0 (0%)	162 (27%)	

CTR, consolidation tumor ratio; SDNs, solid nodules; mGGNs, mixed ground-glass nodules.

**Table 2:** Univariate and binary logistic regression of clinical factors between p-LUSC and SDNs of p-LUAD.

Factors	Univariate Analyses			Binary Logistic Regression	
	p-LUSC, *n* = 103	SDNs of p-LUAD, *n* = 216	*p* Value	Odds Ratio (95%CI)	*p* Value
Male (Female) ^a^	89 (86.4%)	117 (54.2%)	<0.001	6.187 (2.866–13.354)	<0.001
Median age (range), years	68 (47–84)	63 (34–83)	<0.001	1.117 (1.061–1.176)	<0.001
Tumor size (0–2.0 cm)	53 (51.5%)	127 (58.8%)	0.22	-	-
Smoking history (No) ^b^	57 (55.3%)	59 (27.3%)	<0.001	2.506 (1.330–4.723)	0.004
High surgical risk (No) ^b^	38 (36.9%)	48 (22.2%)	0.0058	1.940 (0.977–3.852)	0.058
Ferritin (ng/mL), Median (range) ^c^, % > 322	31.7 (27.3–34.9) 5.8%	31.8 (28.3–35.3) 7.4%	0.69 ^d^	-	-
CEA (ng/mL), Median (range) ^c^, % > 5	2.0 (1.7–2.5) 4.9%	2.0 (1.6–2.3) 4.2%	0.06 ^d^	1.000 (0.845–1.182)	0.996
CA199 (U/mL), Median (range) ^c^, % > 37	21.9 (17.6–26.8) 8.7%	20.6 (16.3–26.0) 6.0%	0.13 ^d^	-	-
CYFRA21-1(ng/mL), Median (range) ^c^, % > 3.3	2.0 (1.5–3.7) 28.2%	1.8 (1.3–2.6) 17.6%	0.024 ^d^	1.272 (0.989–1.636)	0.061
Spicular sign (No)	52 (50.5%)	116 (53.7%)	0.59	-	-
Lobulation sign (No) ^b^	76 (73.8%)	67 (31.0%)	<0.001	6.879 (3.602–13.138)	<0.001
Air bronchogram (No)	32 (31.1%)	64 (29.6%)	0.79	-	-
Pleural indentation (No)	34 (33.0%)	84 (38.9%)	0.31	-	-
Emphysema (No) ^b^	15 (14.6%)	8 (3.7%)	<0.001	2.781 (0.831–9.312)	0.097
Interstitial pneumonia (No) ^b^	25 (24.3%)	19 (8.8%)	<0.001	3.051 (1.197–7.781)	0.019

^a^ Female sex used as reference. ^b^ Negative result used as reference. ^c^ Interquartile range, 25–75%. ^d^ The *p*-value was calculated using the Mann–Whitney U test. CI, Confidence interval.

**Table 3:** Analysis of the relationship between tumor pathological types and malignant invasiveness.

Factors	0–2.0 cm, *n* = 180			>2.0 to ≤3.0 cm, *n* = 139		
	p-LUSC, *n* = 53	SDNs of p-LUAD, *n* = 127	*p* Value	p-LUSC, *n* = 50	SDNs of p-LUAD, *n* = 89	*p* Value
Lymph node metastasis			0.74			<0.001
Positive	3 (5.7%)	9 (7.1%)		3 (6%)	37 (41.6%)	
Negative	50 (94.3%)	118 (92.9%)		47 (94%)	52 (58.4%)	
Pleural invasion			0.0036			<0.001
Positive	2 (3.8%)	27 (21.3%)		3 (6%)	43 (48.3%)	
Negative	51 (96.2%)	100 (78.7%)		47 (94%)	46 (51.7%)	
Lymphovascular invasion			0.45			0.66
Positive	8 (15.1%)	14 (11.0%)		10 (20%)	31 (34.8%)	
Negative	45 (84.9%)	113 (89.0%)		40 (80%)	58 (65.2%)	

**Table 4:** Analysis of the relationship between tumor size and malignant invasiveness.

Factors	p-LUSC, *n* = 103			SDNs of p-LUAD, *n* = 216		
	0–2.0 cm, *n* = 53	>2.0 to ≤3.0 cm, *n* = 50	*p* Value	0–2.0 cm, *n* = 127	>2.0 to ≤3.0 cm, *n* = 89	*p* Value
Lymph node metastasis			0.94			<0.001
Positive	3 (5.7%)	3 (6%)		9 (7.1%)	37 (41.6%)	
Negative	50 (94.3%)	47 (94%)		118 (92.9%)	52 (58.4%)	
Pleural invasion			0.60			<0.001
Positive	2 (3.8%)	3 (6%)		27 (21.3%)	43 (48.3%)	
Negative	51 (96.2%)	47 (94%)		100 (78.7%)	46 (51.7%)	
Lymphovascular invasion			0.51			<0.001
Positive	8 (15.1%)	10 (20%)		14 (11.0%)	31 (34.8%)	
Negative	45 (84.9%)	40 (80%)		113 (89.0%)	58 (65.2%)	

**Table 5:** Univariate and multivariate analyses of prognosis factors.

Factors (Reference)	Univariable		Multivariable	
	HR (95% CI)	*p*-Value	HR (95% CI)	*p*-Value
Male (Female) ^a^	0.877 (0.448–1.715)	0.701	-	-
Age	1.003 (0.962–1.046)	0.882	-	-
Smoker (No) ^b^	1.456 (0.768–2.760)	0.250	-	-
High surgical risk (No) ^b^	0.642 (0.283–1.459)	0.290	-	-
Size > 2.0 to ≤3.0 cm (0–2.0 cm) ^b^	3.507 (1.738–7.076)	<0.001	2.430 (1.135–5.204)	0.022
Lymph node metastasis (No) ^b^	3.924 (2.046–7.524)	<0.001	2.305 (1.083–4.903)	0.030
P-LUSC (p-LUAD) ^c^	0.883 (0.438–1.782)	0.729	-	-
Ferritin, ng/mL	1.003 (1.001–1.005)	0.003	1.001 (0.999–1.003)	0.270
AFP, IU/mL	0.787 (0.522–1.187)	0.254	-	-
CEA, ng/mL	1.115 (0.972–1.278)	0.120	-	-
CA199, U/mL	1.003 (0.974–1.003)	0.824	-	-
NSE, ng/mL	0.970 (0.931–1.010)	0.142	-	-
CYFRA21-1, ng/mL	1.101 (0.871–1.391)	0.420	-	-
Spicular sign (No) ^b^	1.210 (0.635–2.305)	0.563	-	-
Lobulation sign (No) ^b^	1.114 (0.589–2.107)	0.739	-	-
Air bronchogram (No) ^b^	1.352 (0.699–2.614)	0.370	-	-
Pleural indentation (No) ^b^	0.948 (0.490–1.835)	0.874	-	-
Emphysema (No) ^b^	1.880 (0.734–4.817)	0.188	-	-
Interstitial pneumonia (No) ^b^	1.660 (0.730–3.773)	0.227	-	-
Pleural invasion (No) ^b^	2.262 (1.180–4.338)	0.014	1.242 (0.603–2.556)	0.557
Lymphovascular invasion (No) ^b^	1.408 (0.684–2.899)	0.354	-	-

^a^ Female used as reference. ^b^ Negative used as reference. ^c^ SDNs of p-LUAD used as reference. HR, Hazard ratio. CI, Confidence interval.

## Data Availability

The raw data supporting the conclusions of this article will be made available by the authors on request.

## Abbreviations

The following abbreviations are used in this manuscript:

LUSC	lung squamous cell carcinoma
p-LUSC	peripheral lung squamous cell carcinoma
p-LUAD	peripheral lung adenocarcinoma
SDNs	solid nodules
mGGNs	mixed ground-glass nodules
NSCLC	non-small cell lung cancer
GGNs	ground-glass nodules
CTR	consolidation tumor ratio
VDT	volume doubling time
CEA	carcinoembryonic antigen
CA19-9	carbohydrate antigen 19-9
CYFRA21-1	cytokeratin 19 fragment
RFS	relapse-free survival
